# Analysis of volatiles from feces of released Przewalski’s horse (*Equus przewalskii*) in *Gasterophilus pecorum* (Diptera: Gasterophilidae) spawning habitat

**DOI:** 10.1038/s41598-021-95162-9

**Published:** 2021-08-02

**Authors:** Ran Zhou, Jianming Yang, Ke Zhang, Yingjie Qi, Wei Ma, Zhenbiao Wang, Make Ente, Kai Li

**Affiliations:** 1 Key Laboratory of Non-Invasive Research Technology for Endangered Species, School of Ecology and Nature Conservation, Beijing Forestry University, Beijing, 100083 China; 2 Xinjiang Research Centre for Breeding Przewalski’s Horse, Urumqi, 831700 Xinjiang China; 3 Xinjiang Kalamaili Ungulate Nature Reserve Management Center, Changji, 831100 Xinjiang China

**Keywords:** Metabolomics, Chemical ecology, Metabolomics, Conservation biology, Animal physiology, Entomology

## Abstract

The absolute dominant species that infests wild population of Przewalski’s horse (*Equus przewalskii*) is *Gasterophilus pecorum*, and feces of released Przewalski’s horse, a habitat odor, plays an important role in mating and ovipositing locations of *G. pecorum*. To screen out unique volatiles for attracting *G. pecorum*, volatiles from fresh feces of released horses at stages of pre-oviposition (PREO), oviposition (OVIP), and post-oviposition (POSO) of *G. pecorum*, and feces with three different freshness states (*i.e.*, Fresh, Semi-fresh, and Dry) at OVIP were collected by dynamic headspace adsorption and determined by automatic thermal desorption GC–MS. Results show that there were significant differences in fecal volatiles within both test conditions. Of the five most abundant volatiles from the five individual samples, the most important volatile was ammonium acetate at OVIP/Fresh, followed by acetophenone (Semi-fresh), toluene (PREO, OVIP and POSO), butanoic acid (OVIP and Semi-fresh), acetic acid (PREO, POSO and Semi-fresh), 1,6-octadiene,3,7-dimethyl-,(*S*)- (PREO, OVIP and POSO), 1,5,9-undecatriene,2,6,10-trimethyl-,(*Z*)- (PREO and Semi-fresh) and caprolactam (all conditions), which seem to be critical substances in oviposition process of *G. pecorum*. The findings may be beneficial to development of *G. pecorum* attractants*,* facilitating prevention and control of infection by *G. pecorum* to released Przewalski’s horse.

## Introduction

*Gasterophilus* spp. are common obligate parasites of equines, among which *Gasterophilus intestinalis* De Geer is the main dominant parasite species of equines in the world^1–3^, while *Gasterophilus pecorum* Fabricius is the absolute dominant parasite species of equines at Kalamaili Ungulate Nature Reserve (KUNR) in Xinjiang, located in an arid desert steppe. In 2001, a reintroduced endangered species, Przewalski’s horse (*Equus przewalskii*), was released into wild in KUNR, exposing an unusually severe problem of *G. pecorum* infection. What is worse, the infection intensity of *G. pecorum* in Przewalski’s horse was about 2.5 times as much as that of Mongolian wild ass (*Equus hemionus*), accounting for more than 90% of total infection intensity of six *Gasterophilus* spp. in the local area. This phenomenon is rare in equine myiasis all over the world^4–6^.

Mature larvae of *Gasterophilus* spp. are discharged from equine body with its feces and pupate to ground. After automatic emergence, adults mate and oviposit in a frantic manner, but they do not feed, thus their life span is less than a week^7,8^ (pp. 110–128). Cogley and Cogley^8^ reported that equine feces have been found to be the mating site of newly emerged *G. intestinalis.* There are nine *Gasterophilus* spp. in the world, and *G. pecorum* are the most special species among them in regard to their biological characteristics. While other species of *Gasterophilus* spp. oviposit on equine hairs^7^, *G. pecorum* oviposits dispersively on tips of needlegrass (*Stipa caucasica* Schmalh)*,* which is the most preferred plant of Przewalski’s horse in KUNR^9,10^. The number of eggs laid per female *G. pecorum* is 1300–2425, higher than those of other species of the genus. Furthermore, the life span of adult *G. pecorum* is only 1–4 days, and its maximal life time is shorter than those of other species of the genus^7^. Przewalski’s horse needs to drink every day, so its activity is limited to a close range near to the water source^11,12^. After drinking, the horses usually stay around the water source with defecating, feeding, rest and other behaviors. This results in high density areas of Przewalski’s horse feces and *G. pecorum* eggs being highly overlapped around the water source, suggesting that feces may play an indirect guide in the oviposition process of *G. pecorum*^10^*.* Therefore, it could be inferred that vicinity of equine feces is the main site for *G. pecorum* vital activities, including pupation, eclosion, mating, and oviposition. Hence, female flies can locate the oviposition site in a short distance, which could reduce unnecessary energy consumption, and enable efficient use of limited energy to complete larger-scale and more scattered oviposition. This special reproductive strategy of *G. pecorum* may be one of the main reasons for its severe infection of Przewalski’s horse.

It has also been reported that female *G. pecorum* could find indirect host odor (i.e., volatiles released by needlegrass on which Przewalski’s horse feed) to lay eggs by searching habitat odor (i.e., volatiles emitted from equine feces) so their eggs could be taken up by equines to the greatest extent, which could increase probability of *G. pecorum* infesting equines^9,10^. Meiners^13^ pointed out that host odor (i.e., smell given off by equines) or indirect host odor was used to locate specific feeding or spawning sites, while habitat odor represented general area where such host odor was most likely to be found. In addition, habitat odor is characterized by a large amount of release. Although this habitat odor is easier to be detected by distant insects, it lacks specificity. Because of these characteristics, the odor can maximize chances of insects to encounter host odor subsequently. Habitat odor could be associated with secretions or excretions of host, such as those associated with dwellings or nests of blood-sucking insects’ hosts^14^. The combination of volatiles from insect host and its feeding plants could be synergistic signals for female parasitoid to select and locate hosts^15^. Habitat odor could indicate presence of hosts, even increase activity or attraction of insects^16,17^. In the two-way competition test between Przewalski’s horse fresh feces and needlegrass at the oviposition stage of *G. pecorum*, female flies prefer to choose fresh feces. Furthermore, in the four-way competition test, including needlegrass, fresh feces, fresh feces plus needlegrass, and control group, the attraction of female flies to fresh feces plus needlegrass is the strongest. The results of these two competition tests indicate that fresh feces play an important role in oviposition location of *G. pecorum* (Personal communication).

Habitat odor could quickly become an important indicator of host location for host-seeking insects^12^. In the previous study, we have compared volatiles of needlegrass with three test conditions, and found that 3-hexen-1-ol,(*Z*)-, caprolactam, 2(5H)-furanone,5-ethyl-, acetic acid, and hexanal were the five most abundant volatiles of needlegrass at the oviposition stage of *G. pecorum*^18^. In the current work, we analyzed and compared volatiles responsible for odors of Przewalski’s horse feces with different freshness states at the *G. pecorum* oviposition stage as well as those from its fresh feces at the pre-oviposition, oviposition, and post-oviposition stages of *G. pecorum*, and screened out common and unique volatiles of fresh or semi-fresh feces at the oviposition stage of *G. pecorum*. It is inferred that specific volatiles of fresh or semi-fresh feces at the oviposition stage of *G. pecorum* might be key volatiles of fecal odor that can attract *G. pecorum*. The findings of this study can facilitate development of a highly effective attractant that combines fecal odor and grass odor to trap ovipositing *G. pecorum*.

## Results

### The volatiles from fresh feces of Przewalski’s horse at the pre-oviposition, oviposition, and post-oviposition stages of *G. pecorum*

**Figure 1:**
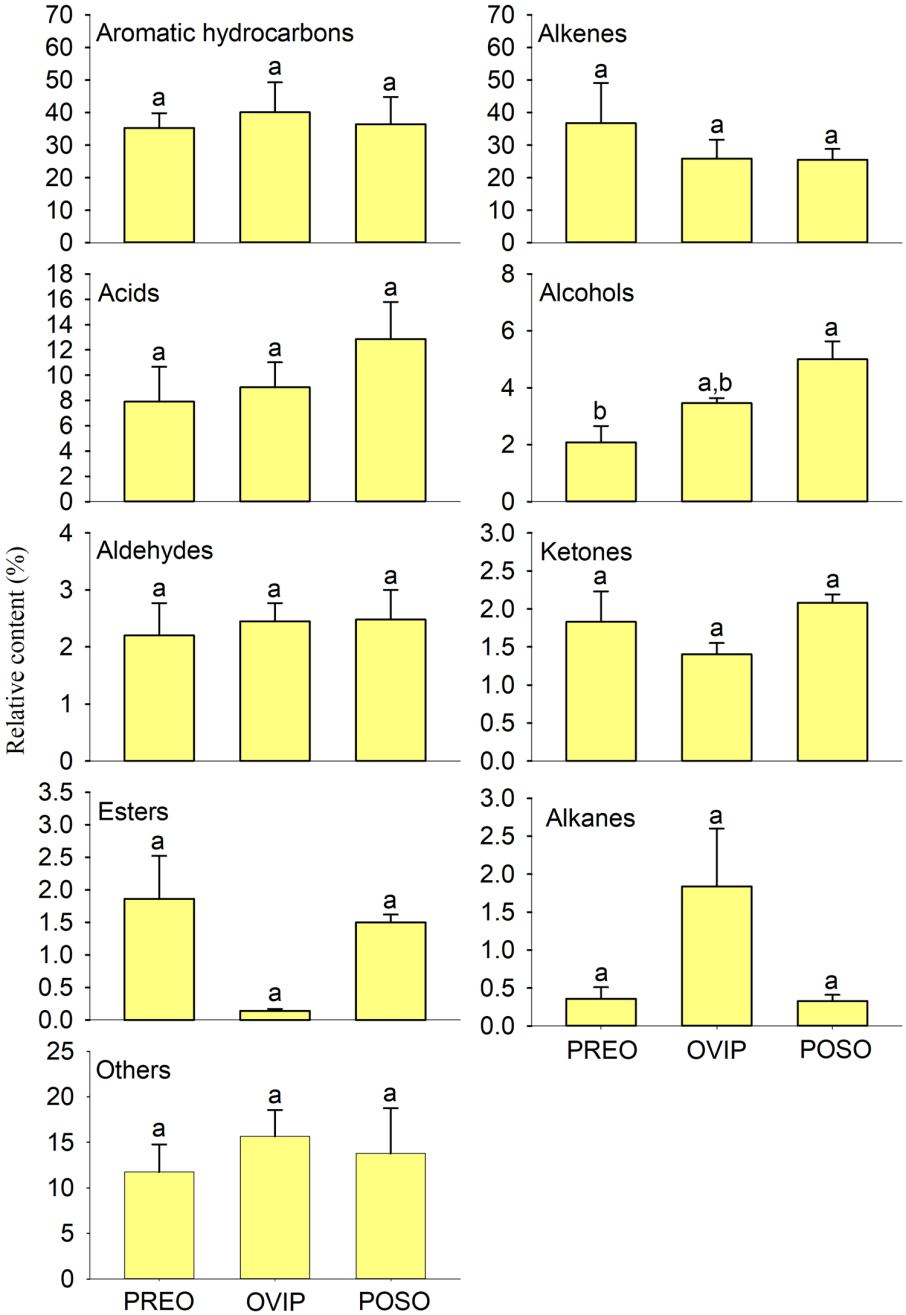
Volatile classes detected from fresh feces of Przewalski’s horse at the stages of PREO, OVIP, and POSO of *Gasterophilus pecorum.* PREO, OVIP, and POSO represent fresh feces at the stages of pre-oviposition, oviposition, and post-oviposition of *Gasterophilus pecorum*, respectively. Data are mean (n = 3) ± SE. Different letters indicate significant differences at *P* < 0.05.

**Figure 2:**
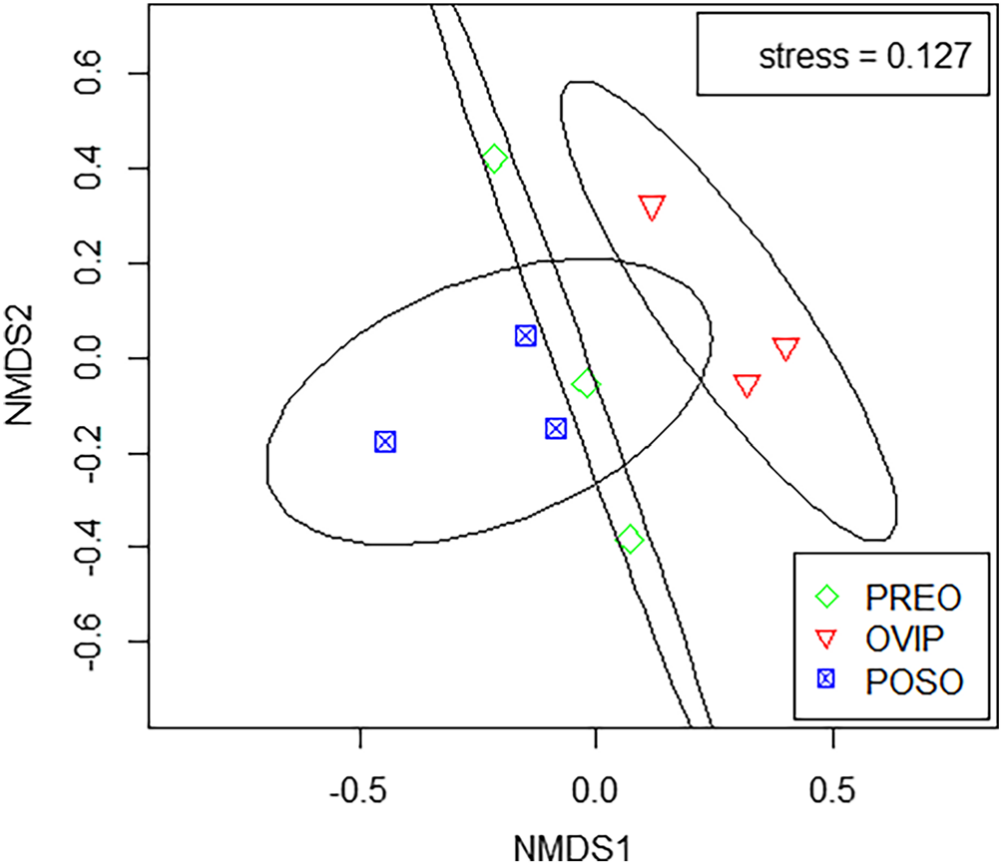
Multivariate analysis of the volatiles from fresh feces of Przewalski’s horse at the stages of PREO, OVIP, and POSO of *Gasterophilus pecorum* by the non-metric multidimensional scaling (NMDS) method. PREO, OVIP, and POSO represent fresh feces at the stages of pre-oviposition, oviposition, and post-oviposition of *Gasterophilus pecorum*, respectively.

Throughout the stages of pre-oviposition (PREO), oviposition (OVIP), and post-oviposition (POSO) of *G. pecorum*, 70 volatiles were identified in fresh feces of Przewalski’s horse. Among them, 46, 48, and 52 volatiles were identified at PREO, OVIP, and POSO, respectively, and 29 volatiles were common at all three stages. In addition, 4, 5, and 9 volatiles were common between PREO and OVIP, OVIP and POSO, as well as PREO and POSO, whereas 4, 10, and 9 volatiles were unique at the single stage of PREO, OVIP, and POSO, respectively (Table 1; Fig. S1). According to relative content, the two main chemical classes of volatiles were aromatic hydrocarbons and alkenes, that is, their respective contents in a sample were both more than 25% of the total content. Except alcohols which exhibited significant difference between PREO and POSO (One-way ANOVA, F = 8.400, *df* = 2, *P* = 0.018), there was no significant difference in all other pairwise comparisons among the nine chemical classes at three stages (One-way ANOVA or Kruskal–Wallis test: *P* > 0.05) (Fig. 1). Non-metric multidimensional scaling (NMDS) analysis revealed certain extent of overlap (Fig. 2), while one-way analysis of similarity (ANOSIM) indicated that there were significant differences among the three stages (R = 0.5391, *P* = 0.008).

**Table 1:** The volatiles from fresh feces of Przewalski’s horse at the stages of PREO, OVIP, and POSO of *Gasterophilus pecorum.*

Chemical Class	Compound	PREO	OVIP	POSO
Aromatic hydrocarbons	Toluene*	35.19 ± 4.57a **	40.03 ± 9.18a	33.09 ± 8.77a
	1H-Indene,1-methylene-	0.07 ± 0.02a	0.08 ± 0.02a	0.09 ± 0.01a
	*Benzene,2-propenyl-*	–	–	3.23 ± 0.56
Alkenes	1,7-Octadiene,2,7-dimethyl-	0.77 ± 0.32a	2.17 ± 0.96a	2.18 ± 0.59a
	*1,6-Octadiene,3,7-dimethyl-,(S)-*	18.09 ± 11.73a	11.98 ± 2.24a	15.65 ± 3.90a
	Camphene	0.90 ± 0.09a	0.64 ± 0.12a	0.99 ± 0.39a
	Thujene	–	0.21 ± 0.02	–
	π-Pinene	0.63 ± 0.28a	0.50 ± 0.07a	–
	Cyclohexene,3-methyl-6-(1-methylethyl)-	–	3.26 ± 1.67	–
	1,3-Methanopentalene,1,2,3,5-tetrahydro-	–	0.21 ± 0.01	–
	*D*-limonene***	2.95 ± 0.47a	0.87 ± 0.28b	1.13 ± 0.14b
	π-Phellandrene	2.02 ± 0.84a	0.32 ± 0.04a	0.58 ± 0.23a
	1,4-Cyclohexadiene,1-methyl-4-(1-methylethyl)-	2.16 ± 1.16a	2.25 ± 1.34a	1.95 ± 0.30a
	Cyclohexene,1-methyl-4-(1-methylethylidene)-	0.69 ± 0.19a	0.72 ± 0.23a	–
	*1,5,9-Undecatriene,2,6,10-trimethyl-,(Z)-*	6.27 ± 1.32a	2.4 ± 0.76a	–
	(-)-isocaryophyllene	–	0.38 ± 0.11a	0.39 ± 0.07a
	Sabinene	0.47 ± 0.26a	–	0.71 ± 0.11a
	( +)-4-Carene	1.51 ± 0.45a	–	0.57 ± 0.10a
	2-Octene,3,7-dimethyl-,(*Z*)-	–	–	0.21 ± 0.02
	(1S)-(-)-beta-Pinene	–	–	0.96 ± 0.15
	Elixene	0.30 ± 0.08a	–	0.23 ± 0.01a
Acids	Hexanoic acid	–	2.25 ± 0.47	–
	*Butanoic acid*	1.13 ± 0.39a	3.69 ± 1.62a	2.90 ± 0.76a
	Butanoic acid,3-methyl-	0.58 ± 0.22a	1.36 ± 0.57a	1.86 ± 0.84a
	Butanoic acid,2-methyl-	0.61 ± 0.28a	1.74 ± 0.47a	2.11 ± 0.35a
	*Acetic acid*	5.28 ± 1.92a	–	4.98 ± 0.98a
	Propanoic acid	0.30 ± 0.22	–	–
	Propanoic acid,2-methyl-	–	–	0.98 ± 0.64
Alcohols	1-Propanol	0.16 ± 0.06a	0.40 ± 0.13a	0.28 ± 0.09a
	1-Propanol,2-methyl-	–	0.33 ± 0.07	–
	**1-Butanol**	0.32 ± 0.09b	1.12 ± 0.20a	0.52 ± 0.12b
	1-Butanol,3-methyl-	0.27 ± 0.02a	0.69 ± 0.10a	0.84 ± 0.41a
	**1-Butanol,2-methyl-**	0.27 ± 0.05a	0.68 ± 0.03b	–
	Eucalyptol	0.14 ± 0.05a	0.06 ± 0.01a	0.12 ± 0.10a
	Thujol	–	0.03 ± 0.01a	0.06 ± 0.01a
	l-Menthol	–	0.07 ± 0.01a	0.11 ± 0.01a
	**Carvomenthol**	0.12 ± 0.10b	0.08 ± 0.01b	0.5 ± 0.11a
	3,4-Dihydroxybenzyl alcohol,tris(trimethylsilyl)-	0.80 ± 0.27a	–	0.95 ± 0.04a
	6,11-Dimethyl-2,6,10-dodecatrien-1-ol	–	–	1.61 ± 0.19
Aldehydes	Pentanal	1.48 ± 0.44a	1.52 ± 0.15a	1.23 ± 0.36a
	Heptanal	–	0.08 ± 0.01a	0.09 ± 0.00a
	Benzaldehyde	0.13 ± 0.03a	0.13 ± 0.03a	0.22 ± 0.06a
	Octanal	0.16 ± 0.05a	0.25 ± 0.08a	0.22 ± 0.03a
	Nonanal	0.28 ± 0.09a	0.35 ± 0.04a	0.36 ± 0.12a
	Decanal	0.07 ± 0.01a	0.12 ± 0.02a	0.12 ± 0.03a
	2-Heptenal,(*Z*)-	0.08 ± 0.02	–	–
	Butanal,3-methyl-	–	–	0.12 ± 0.03
	3,3-Diethoxy-1-propyne	–	–	0.12 ± 0.02
Ketones	Acetone	0.18 ± 0.03a	0.31 ± 0.05a	0.27 ± 0.06a
	2-Hexanone,4-methyl-	–	0.65 ± 0.10	–
	Acetophenone	0.23 ± 0.07a	0.18 ± 0.06a	0.17 ± 0.07a
	l-Camphor	0.25 ± 0.06a	0.11 ± 0.03a	0.31 ± 0.09a
	Cyclohexanone,5-methyl-2-(1-methylethyl)-,cis-	–	0.71 ± 0.00	–
	5,9-Undecadien-2-one,6,10-dimethyl-,(*E*)-	0.07 ± 0.02a	0.09 ± 0.01a	0.11 ± 0.02a
	2-Heptanone	0.35 ± 0.07	–	–
	5-Hepten-2-one,6-methyl-	0.58 ± 0.15a	–	0.65 ± 0.08a
	Thujone	0.12 ± 0.02a	–	0.12 ± 0.03a
	2-Cyclopentylcyclopentanone	0.05 ± 0.02	–	–
	Cyclohexanone,2-methyl-5-(1-methylethyl)-,trans-	–	–	0.45 ± 0.07
Esters	Bisisobutyric acid 2,4,4-trimethylpentane-1,3-diyl ester	–	0.14 ± 0.03	–
	Ethyl acetate	1.49 ± 0.58a	–	0.28 ± 0.07a
	**2,4,4-Trimethyl-1,3-pentanediol 1-isobutyrate**	0.37 ± 0.08a	–	0.75 ± 0.03b
	1-tert-Butyl-2-methyl-1,3-propanediol 1-isobutyrate	–	–	0.48 ± 0.02
Alkanes	Nonane	0.19 ± 0.10a	0.15 ± 0.03a	0.21 ± 0.06a
	Cyclopentasiloxane,decamethyl-	–	1.62 ± 0.74	–
	Undecane,2,6-dimethyl-	0.17 ± 0.06a	0.07 ± 0.01a	0.12 ± 0.04a
Others	*Ammonium acetate*	–	7.79 ± 0.98	–
	**Dimethyl sulfone**	–	0.12 ± 0.01a	0.40 ± 0.06b
	Phenol,4-methyl-	0.82 ± 0.51a	1.11 ± 0.26a	2.02 ± 0.39a
	*Caprolactam*	10.93 ± 2.94a	6.64 ± 1.85a	11.37 ± 4.64a

PREO, OVIP, and POSO represent fresh feces at the stages of pre-oviposition, oviposition, and post-oviposition of *Gasterophilus pecorum*, respectively. The match values of all substances were greater than 700, and those less than 700 were uncertain compounds, which were not listed in this Table 1. *Volatiles written in italic font indicate those of the five most abundant substances between the fresh feces of different stages. **Data are mean (n = 3) ± SE. Different letters indicate significant differences at *P* < 0.05. ***Volatiles written in bold font indicate those with significant differences between the fresh feces of different stages.

Among the eight volatiles which were the five most abundant volatiles at least at one of the three stages, toluene, 1,6-Octadiene,3,7-dimethyl-,(*S*)-, and caprolactam were identified at all three stages. For each of these three volatiles, its relative contents at three stages revealed no significant differences (One-way ANOVA: *P* > 0.05). Furthermore, acetic acid was common to PREO and POSO, but no difference was observed between them (Independent *t* test, t = 0.137*,* *df* = 4*, P* = 0.897) (Table 1).

Of particular concern among the eight volatiles mentioned above, ammonium acetate and butanoic acid were unique to OVIP, the critical stage of oviposition. Although not one of the five most abundant volatiles, another nine volatiles were also specific to OVIP, of which hexanoic acid, cyclopentasiloxane,decamethyl- and cyclohexene,3-methyl-6-(1-methylethyl)- were higher than 1% in relative content (Table 1).

Among the 47 volatiles common to two or three stages, only six volatiles were significantly different in relative contents. Of which, *D*-limonene was higher at PREO than at OVIP (One-way ANOVA: F = 11.936, *df* = 2, *P* = 0.012) or POSO (*P* = 0.012), and 1-butanol was higher at OVIP than at PREO (One-way ANOVA: F = 8.175, *df* = 2, *P* = 0.024) or POSO (*P* = 0.04). Relative contents of the other four volatiles were less than 1% (Table 1).

### The volatiles from feces of Przewalski’s horse with different freshness states at the OVIP stage of *G. pecorum*

**Figure 3:**
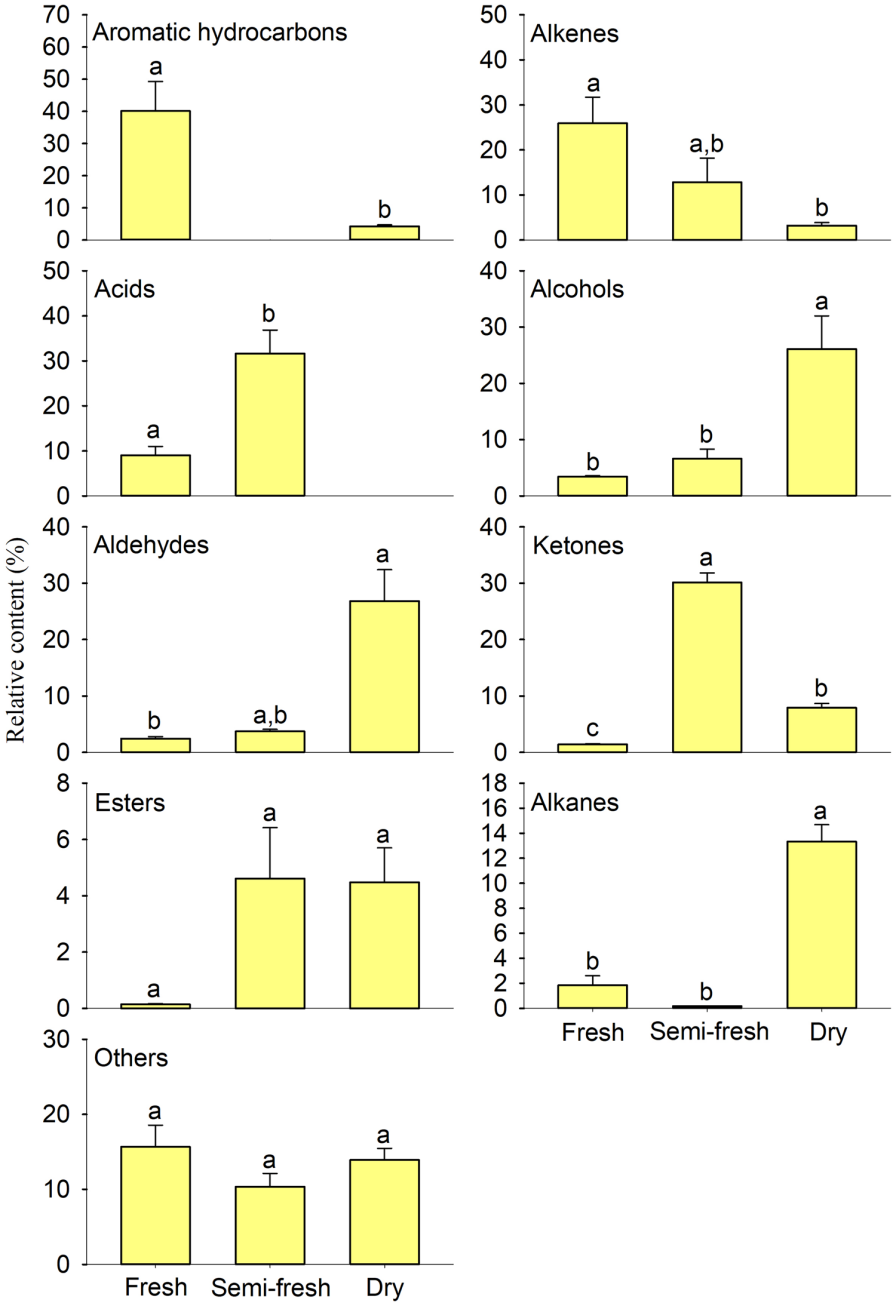
Volatile classes detected from feces of Przewalski’s horse with different freshness states at the OVIP stage of *Gasterophilus pecorum.* Fresh, Semi-fresh, and Dry represent fresh, semi-fresh, and dry feces at the oviposition (OVIP) stage of *Gasterophilus pecorum*, respectively. Data are mean (n = 3) ± SE. Different letters indicate significant differences at *P* < 0.05.

**Figure 4:**
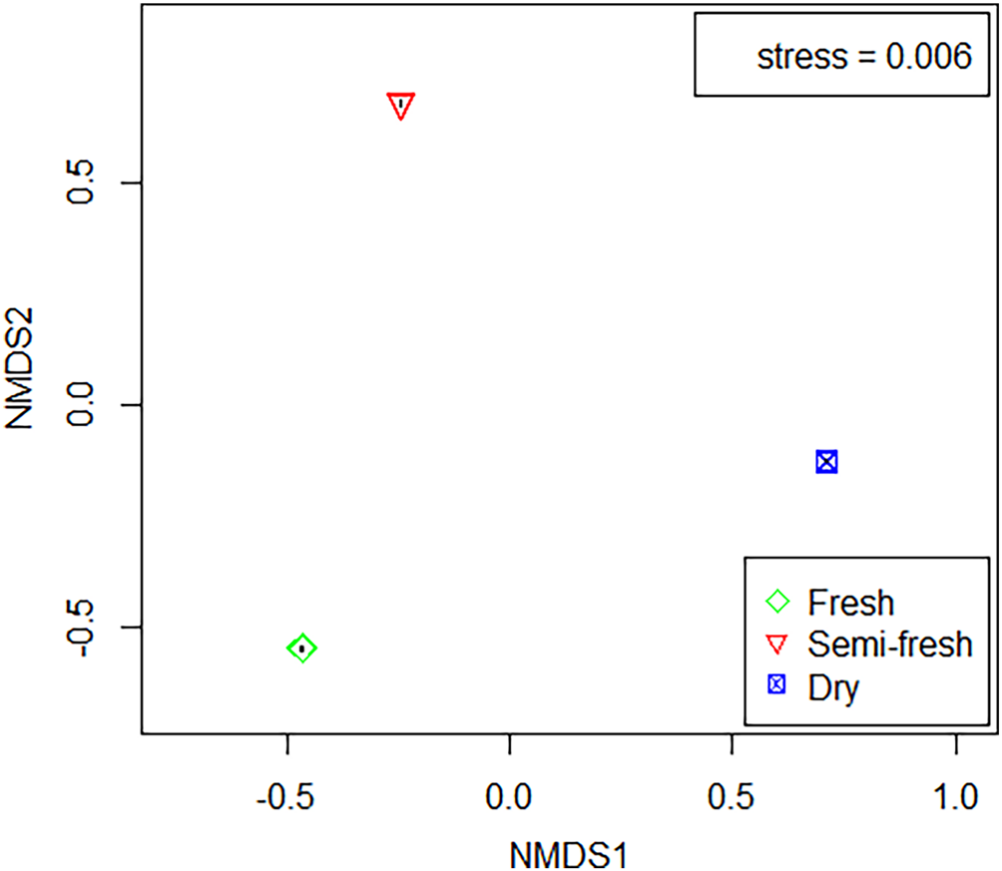
Multivariate analysis of the volatiles from feces of Przewalski’s horse with the three different freshness states at the OVIP stage of *Gasterophilus pecorum* by the non-metric multidimensional scaling (NMDS) method. Fresh, Semi-fresh, and Dry represent fresh, semi-fresh, and dry feces at the oviposition (OVIP) stage of *Gasterophilus pecorum*, respectively.

Totally, 83 volatiles were detected from fresh feces (Fresh), semi-fresh feces (Semi-fresh), and dry feces (Dry) at the OVIP stage of *G. pecorum*. Of which, 48, 41 and 28 volatiles were identified in Fresh, Semi-fresh and Dry, and 7 volatiles were common to all three feces with different freshness states. In addition, 14, 3 and 3, were common between Fresh and Semi-fresh, Semi-fresh and Dry, as well as Fresh and Dry, whereas 24, 17, and 15 were unique to Fresh, Semi-fresh, and Dry, respectively (Table 2; Fig. S2). Aromatic hydrocarbons and alkenes, acids and ketones, as well as alcohols and aldehydes were the two main chemical classes of Fresh, Semi-fresh, and Dry in respective. Except esters and ‘others’ which showed no significant difference in the feces, there were significant differences among other seven classes in at least one pairwise comparison of the three freshness states (One-way ANOVA, Independent *t*-test or Kruskal–Wallis test: *P* < 0.05) (Fig. 3). NMDS analysis revealed no overlap among the three states, and noticeably, three data points per states were all identical (Fig. 4). Moreover, ANOSIM indicated there were significant differences among the three states (R = 1, *P* = 0.005).

**Table 2:** The volatiles from feces of Przewalski’s horse with different freshness states at the OVIP stage of *Gasterophilus pecorum.*

Chemical Class	Compound	Fresh	Semi-fresh	Dry
Aromatic hydrocarbons	Toluene*	40.03 ± 9.18	–	–
	1H-Indene,1-methylene-***	0.08 ± 0.02a**	–	1.19 ± 0.19b
	p-Xylene	–	–	1.64 ± 0.33
	Benzene,1-ethyl-3-methyl-	–	–	0.71 ± 0.13
	1H-Indene,1-ethylidene-	–	–	0.69 ± 0.11
Alkenes	1,7-Octadiene,2,7-dimethyl-	2.17 ± 0.96a	0.77 ± 0.28a	–
	*1,6-Octadiene,3,7-dimethyl-, (S)-*	11.98 ± 2.24a	4.86 ± 1.70a	–
	Camphene	0.64 ± 0.12	–	–
	Thujene	0.21 ± 0.02	–	–
	π-Pinene	0.50 ± 0.07	–	–
	Cyclohexene,3-methyl-6-(1-methylethyl)-	3.26 ± 1.67	–	–
	1,3-Methanopentalene,1,2,3,5-tetrahydro-	0.21 ± 0.01a	0.34 ± 0.09a	–
	*D*-Limonene	0.87 ± 0.28	–	–
	π-Phellandrene	0.32 ± 0.04	–	–
	1,4-Cyclohexadiene,1-methyl-4-(1-methylethyl)-	2.25 ± 1.34	–	–
	Cyclohexene,1-methyl-4-(1-methylethylidene)-	0.72 ± 0.23	–	–
	*1,5,9-Undecatriene,2,6,10-trimethyl-,(Z)-*	2.40 ± 0.76a	6.50 ± 3.50a	–
	(-)-isocaryophyllene	0.38 ± 0.11	–	–
	**Limonene**	–	0.30 ± 0.02a	2.40 ± 0.56b
	1,3-Cyclopentadiene,5-(1-methylethylidene)-	–	–	0.80 ± 0.17
Acids	Hexanoic acid	2.25 ± 0.47	–	–
	***Butanoic acid***	3.69 ± 1.62a	12.08 ± 2.26b	–
	Butanoic acid,3-methyl-	1.36 ± 0.57a	4.92 ± 1.28a	–
	Butanoic acid,2-methyl-	1.74 ± 0.47	–	–
	*Acetic acid*	–	11.46 ± 0.43	–
	Propanoic acid,2-methyl-	–	3.17 ± 1.98	–
Alcohols	1-Propanol	0.40 ± 0.13	–	–
	1-Propanol,2-methyl-	0.33 ± 0.07	–	–
	1-Butanol	1.12 ± 0.20a	0.98 ± 0.22a	–
	1-Butanol,3-methyl-	0.69 ± 0.10	–	–
	1-Butanol,2-methyl-	0.68 ± 0.03	–	–
	Eucalyptol	0.06 ± 0.01	–	–
	Thujol	0.03 ± 0.01	–	–
	l-Menthol	0.07 ± 0.01a	–	1.18 ± 0.67a
	Carvomenthol	0.08 ± 0.01a	0.37 ± 0.24a	–
	***3,4-Dihydroxybenzyl alcohol,tris(trimethylsilyl)-***	–	0.82 ± 0.0.27a	24.92 ± 6.35b
	1-Pentanol	–	4.44 ± 1.01	–
Aldehydes	Pentanal	1.52 ± 0.15	–	–
	**Heptanal**	0.08 ± 0.01c	1.03 ± 0.15b	2.69 ± 0.26a
	Benzaldehyde	0.13 ± 0.03a	0.10 ± 0.07a	0.60 ± 0.17a
	**Octanal**	0.25 ± 0.08b	0.43 ± 0.03b	2.22 ± 0.38a
	***Nonanal***	0.35 ± 0.04c	0.90 ± 0.16b	11.74 ± 3.19a
	***Decanal***	0.12 ± 0.02b	0.34 ± 0.12b	5.49 ± 2.18a
	2-Heptenal,(*Z*)-	–	0.20 ± 0.04	–
	2-Hexenal	–	0.40 ± 0.09	–
	2-Nonenal,(*E*)-	–	0.18 ± 0.04	–
	1-Cyclohexene-1-carboxaldehyde,2,6,6-trimethyl-	–	0.14 ± 0.04	–
	Hexanal	–	–	4.10 ± 0.32
Ketones	Acetone	0.31 ± 0.05a	0.50 ± 0.05a	–
	2-Hexanone,4-methyl-	0.65 ± 0.10	–	–
	***Acetophenone***	0.18 ± 0.06a	12.35 ± 3.93b	–
	l-Camphor	0.11 ± 0.03a	0.27 ± 0.16a	–
	Cyclohexanone,5-methyl-2-(1-methylethyl)-,cis-	0.71 ± 0.00	–	–
	**5,9-Undecadien-2-one,6,10-dimethyl-,(*E*)-**	0.09 ± 0.01c	0.37 ± 0.13b	1.21 ± 0.24a
	**5-Hepten-2-one,6-methyl-**	–	1.40 ± 0.12a	4.02 ± 0.56b
	2-Pentanone	–	1.34 ± 0.36	–
	2-Butanone,3-hydroxy-	–	2.94 ± 0.76	–
	2-Pentanone,3-methyl-	–	0.47 ± 0.05	–
	2-Hexanone	–	4.51 ± 1.01	–
	2-Hexanone,5-methyl-	–	1.41 ± 0.31	–
	2-Heptanone,6-methyl-	–	0.20 ± 0.05	–
	2-Heptanone,5-methyl-	–	1.08 ± 0.25	–
	2-Octanone	–	2.33 ± 0.52	–
	2-Nonanone	–	0.96 ± 0.24	–
	5,9-Dodecadien-2-one,6,10-dimethyl-,(*E,E*)-	–	–	1.60 ± 0.42
	2-Pentadecanone,6,10,14-trimethyl-	–	–	1.09 ± 0.48
Esters	**Bisisobutyric acid 2,4,4-trimethylpentane-1,3-diyl ester**	0.14 ± 0.03a	–	0.67 ± 0.13b
	Ethyl acetate	–	–	3.81 ± 1.34
	Ethanol,2-nitro-,propionate (ester)	–	4.61 ± 1.81	–
Alkanes	Nonane	0.15 ± 0.03	–	–
	Cyclopentasiloxane,decamethyl-	1.62 ± 0.74	–	–
	**Undecane,2,6-dimethyl-**	0.07 ± 0.01a	0.17 ± 0.03b	–
	Hexane	–	–	1.79 ± 0.23
	Cyclotrisiloxane,hexamethyl-	–	–	1.19 ± 0.28
	*Decane,2,2-dimethyl-*	–	–	4.64 ± 1.29
	Decane	–	–	3.32 ± 0.53
	2,2,4,4-Tetramethyloctane	–	–	1.51 ± 0.37
	Cyclopentane,pentyl-	–	–	0.86 ± 0.16
Others	*Ammonium acetate*	7.79 ± 0.98	–	–
	**Dimethyl sulfone**	0.12 ± 0.01a	0.69 ± 0.16b	–
	Phenol,4-methyl-	1.11 ± 0.26a	1.86 ± 0.33a	–
	*Caprolactam*	6.64 ± 1.85a	7.79 ± 1.78a	13.66 ± 1.47a
	2,3-Heptadien-5-yne,2,4-dimethyl-	–	–	0.25 ± 0.10

Fresh, Semi-fresh, and Dry represent fresh, semi-fresh, and dry feces at the oviposition (OVIP) stage of *Gasterophilus pecorum*, respectively. The match values of all substances were greater than 700, and those less than 700 were uncertain compounds, which were not listed in this Table 2. *Volatiles written in italic font indicate those of the five most abundant substances between the feces with different freshness. **Data are mean (n = 3) ± SE. Different letters indicate significant differences at *P* < 0.05. ***Volatiles written in bold font indicate those with significant differences between the feces with different freshness.

Of the twelve volatiles which were one of the five most abundant volatiles at least in one state, only caprolactam occurred in all three states, which was not significantly different among Fresh, Semi-fresh and Dry (One-way ANOVA: F = 4.876, *df *= 2, *P* > 0.05). Besides, butanoic acid was common to Semi-fresh and Fresh, and its level in the former was higher than that in the latter (Independent *t*-test, t = *-*3.018*,* *df* = 4*, P* = 0.039) (Table 2).

It is worthy to note that toluene, 1,6-octadiene,3,7-dimethyl-,(*S*)-, and ammonium acetate were unique to Fresh (i.e., OVIP), and acetophenone, acetic acid, and 1,5,9-undecatriene,2,6,10-trimethyl-,(*Z*)- were unique to Semi-fresh among the twelve volatiles mentioned above. Although not one of the five most abundant volatiles, another twenty-two volatiles were also unique to Fresh, of which cyclohexene,3-methyl-6-(1-methylethyl)-, butanoic acid,2-methyl-, 1,4-cyclohexadiene,1-methyl-4-(1-methylethyl)-, hexanoic acid, cyclopentasiloxane,decamethyl- and pentanal were higher than 1% in relative content. Similarly, another sixteen volatiles were also unique to Semi-fresh, of which propanoic acid,2-methyl-, 1-pentanol, 2-pentanone, 2-butanone,3-hydroxy-, 2-hexanone, 2-hexanone,5-methyl-, 2-heptanone,5-methyl-, 2-octanone and ethanol,2-nitro-,propionate (ester) were higher than 1% (Table 2).

Among the 27 volatiles common to two or three states, except for butanoic acid and acetophenone which belonged to the five most abundant volatiles, only twelve volatiles were significantly different in relative content in at least one pairwise comparison of the three states. Of which, 1H-indene,1-methylene- was higher in Dry than in Fresh (Independent *t*-test, t = *−* 9.288*,* *df* = 4*, P* = 0.001), limonene was higher in Dry than in Semi-fresh (Independent *t*-test, t = *−* 3.747*,* *df* = 4, *P* = 0.020), so was 3,4-Dihydroxybenzyl alcohol,tris(trimethylsilyl)- (Independent *t*-test, t = *−* 9.007*,* *df* = 4*, P* = 0.001). Nonanal and heptanal were higher in Dry than in Semi-fresh (One-way ANOVA: *P* < 0.001 and *P* = 0.002) or Fresh (*P* < 0.001 and 0.001) with significant differences between Semi-fresh and Fresh (*P* = 0.030 and 0.009). Decanal and octanal were higher in Dry than in Semi-fresh (One-way ANOVA:* P* = 0.042 and 0.002) or Fresh (*P* = 0.042 and 0.002). 5,9-Undecadien-2-one,6,10-dimethyl-,(*E*)- was higher in Dry than in Semi-fresh (One-way ANOVA: F = 28.826, *df* = 2, *P* = 0.011) or Fresh (*P* < 0.001), with significant difference between the latter two (*P* = 0.011). 5-Hepten-2-one,6-methyl- was higher in Dry than in Semi-fresh (Independent *t*-test, t = *−* 4.545*,* *df* = 4*, P* = 0.010). Relative content of other three volatiles were less than 1% (Table 2).

## Discussion

Our results showed that the main chemical classes in fresh feces at all PREO, OVIP, and POSO stages of *G. pecorum* were the same, but they were totally different to those detected in a previous study from needlegrass at three equivalent stages^18^. In addition, the number of volatiles in fresh feces were higher than those in needlegrass at equivalent three stages^18^. These differences of volatiles between needlegrass and feces exist because plant volatiles were synthesized by its secondary metabolic pathways^19^, while fecal volatiles were transformed via microbial degradation from carbohydrates, lipids, and proteins of horse’s forage^20^. Furthermore, different combinations of main volatile classes between feces and needlegrass at the OVIP stage could make volatile gradients obvious between them, which might be helpful for *G. pecorum* to oviposit efficiently on tips of needlegrass within range of equines, allowing *G. pecorum* eggs to be easily ‘eaten’ by equines.

We found less volatiles in Dry than in Semi-fresh and even less than in Fresh, and their main chemical classes were very different. The environmental factors of temperature, precipitation, and solar radiation etc., could accelerate or reduce fecal decomposition^21^. Albuquerque and Zurek^22^ reported that decomposition of equine feces could be accelerated when temperature was higher than 26 °C. Our results suggested that main volatile classes changed and volatile amounts decreased with the decrease of fecal moisture contents, especially in the case of Dry.

Toluene was among the five most abundant volatiles, although there existed no significant difference among three fresh feces at PREO, OVIP, and POSO, it was only found in Fresh but not in Semi-fresh and Dry. In dry season, toluene is the most important volatile released from feces of non-territorial male white rhino^23^. Furthermore, it is also one of the main volatiles of swine manure, and its concentration is kept stable during swine manure biogas digestate storage^24^. Saddler^25^ reported that different doses of toluene could induce the electroantennogram (EAG) responses of stable fly (*Stomoxys calcitrans* L.) and house fly (*Musca domestica* L.), with that of the former being stronger than that of the latter at most doses*.* 1,6-Octadiene,3,7-dimethyl-,(*S*)-, toluene, and 1,5,9-undecatriene,2,6,10-trimethyl-,(*Z*)- were already found in white rhino feces^26^. In our study, 1,6-Octadiene,3,7-dimethyl-,(*S*)- was one of the five most abundant volatiles, and it was detected in feces of different conditions except in Dry.

Butanoic acid was only found in the five most abundant substances of Fresh and Semi-fresh, with significant differences between them. Acetic acid was only found in the five most abundant volatiles of PREO, POSO, and Semi-fresh, with no significant differences between PREO and POSO, whereas relative content of acetic acid in Semi-fresh was 3.41 times higher than that of needlegrass at the OVIP stage^18^. In addition, butanoic acid and acetic acid from different sources were verified in EAG responses of many insects. Robinson et al*.*^27^ found that butanoic acid from human feces induced EAG response of bazaar fly (*Musca sorbens* Wiedemann), while that in steers’ rumens volatiles could attract stable fly^28^. Butanoic acid found in pig manures is one of the most active compounds that induce EAG response of female house fly, but acetic acid does not induce the same response^29^. Moreover, EAG responses of stable fly to these two acids are not significantly different from the control treatments (dichloromethane)^30^. Butanoic acid is electrophysiological active on antennae of female blow fly (*Calliphora vicina* R.­D.), but acetic acid is not^31^. In addition, several studies have shown that acetic acid, which is the main composition of blue orchard bee (*Osmia lignaria* Say) frass, could attract two parasitoids (*Monodontomerus torchioi* Grissell and *Melittobia acasta* Walker)^32,33^, and EAG response of *Oedemera virescens* L. is evoked by acetic acid^34^. The sensibility of female house flies to acetic acid is higher than that of males, but it is found that acetic acid has excitatory or inhibitory effects on antennal cells of mature female and male house flies^35^.

Acetophenone was found only in the five most abundant substances of Semi-fresh. Jeanbourquin and Guerin^28^ reported that acetophenone, found in steer’s rumens and derived from L-phenylalanine, was a chemical stimulus for stable fly. Jonfia-Essien et al*.*^36,37^ found that acetophenone had a positive effect on the growth and development of red flour beetle (*Tribolium castaneum* Herbst) and cigarette beetle (*Lasioderma serricorne* Fabricius), and it was also an active substance stimulating antennae of red flour beetle. Jeanbourquin and Guerin^20^ reported that EAG response of stable fly to acetophenone from equine feces was stronger than that from cattle feces, in which low levels of acetophenone were detected. Paczkowski et al.^31^ found that acetophenone evoked electrophysiological activity on female blow fly antennae. Additionally, among all volatiles detected, caprolactam was one of the five most abundant substances in feces of all different samples, but its relative content was significantly higher in Dry than in Fresh. Furthermore, the relative contents of caprolactam in fresh feces were lower than those from needlegrass at three equivalent stages of PREO, OVIP, and POSO^18^.

On the whole, in the five most abundant substances of feces within both test conditions, ammonium acetate was only found in Fresh/OVIP. Ammonia is an indicator to inform female fruit flies about the presence of protein needed for ovipositing, which could derive from protein hydrolysates, ammonium salts, fertilizers, and feces^38–40^, so these substances could be used as an attractant for female fruit fly. Among them, ammonium acetate is the most effective attractant of various ammonium salts, and also widely used as attractant of fruit flies^41^. Mazor^41^ further indicated that ammonium acetate, as the source of ammonia and acetic acid, was more attractive to male Mediterranean fruit flies (*Ceratitis capitata*) than to its females, whereas acetic acid was barely attractive to females and very attractive to males. Piñero et al*.*^42^ reported that protein baits supplemented with ammonium acetate could significantly increase attraction of female Mediterranean fruit fly*.* Our recent study also found that attraction of Fresh to *G. pecorum* was the most effective, followed by Semi-fresh, but that of Dry was not obvious (Personal communication). Although adult *G. pecorum* do not feed, the study indicated ammonium acetate was unique to Fresh, so this volatile must be further investigated in relation to EAG responses of *G. pecorum* in the future.

Several studies have shown that strong fecal odor was usually associated with newly deposited wet but not dry feces^43^. Our results show that volatiles common to Fresh, Semi-fresh and Dry were low, and a great difference in the number of volatiles among three states was observed. Cooperband et al*.*^44^ reported that fresh chicken feces were attractive to southern house mosquito (*Culex quinquefasciatus* Say), but non-fresh and dried chicken feces were not, indicating that fresh chicken feces emit attractive volatiles which indicate recent existence of their host, but non-fresh feces provide less useful information in this respect. However, Albuquerque and Zurek^22^ reported that fresh horse feces did not stimulate oviposition of stable fly, but increased attraction in connection with aging of feces until 3 weeks of age. Schlein et al*.*^45^ suggested that aging cattle feces increased field trapping rates of female and male *Phlebotomus papatasi* Scopoli, which could be related to the search of oviposition sites and mates.

Indole is a long-range attractant with moderate volatility, while more volatile alcohols can be used as a short-range attractant to help blowflies locate feces accurately^42^. In the previous study, we have indicated that relative content of alcohols from needlegrass at the oviposition stage of *G. pecorum* was the highest^18^, whereas that from fresh feces at the same stage was lower in this study, which could associate with short-range and long-range attractant usage. In addition, in the present study, oviposition stage of *G. pecorum* was very short, thus only three replicates were made for each sample. The study is a starting point for the development of attractants for *G. pecorum*, and the sample size should be increased for further investigations.

Habitat odor was found to change in conjunction with host odor, probably enhancing the behavioral response of insects by providing important background information^46^. Habitat odor could make insects sensitive to host volatiles and enhance their responses to host odor^46^. In the absence of habitat odor, the responses to host odors detected by insects are either weak or ignored^14^. Of the five most abundant volatiles from the five individual samples, the most important volatile was ammonium acetate at OVIP/Fresh, followed by acetophenone (Semi-fresh), toluene (PREO, OVIP and POSO), butanoic acid (OVIP and Semi-fresh), and acetic acid (PREO, POSO and Semi-fresh). Hence, these five volatiles deserve special attention and it should be tested whether they evoke EAG responses or attract *G. pecorum* in follow-up studies*.* Although there was no report on roles of 1,6-octadiene,3,7-dimethyl-,(*S*)- (PREO, OVIP and POSO), 1,5,9-undecatriene,2,6,10-trimethyl-,(*Z*)- (PREO and Semi-fresh) and caprolactam (all conditions) in EAG responses or attraction of insects, they were also one of the five most abundant volatiles, so they should also be paid attention to in subsequent studies. Therefore, characterizations of fecal odor (volatiles of Fresh and Semi-fresh) in the current study and grass odor (volatiles released by needlegrass) in our previous study^18^ at the OVIP stage of *G. pecorum* would provide basic theoretical data for development of its effective attractant. Based on this, in the next study, we can fence the common water source of Przewalski’s horse and artificially place attractants at the enclosed water source to attract female *G. pecorum* to lay eggs at its OVIP stage, so as to further explore the molecular mechanism of the key fecal and grass volatiles in its oviposition location.

## Materials and methods

### Ethical statement

The study was carried out in accordance with the Chinese laws and regulations of the Beijing Forestry University. The experimental protocol was reviewed and approved by the Ethic and Animal Welfare Committee of Beijing Forestry University, and no animals were harmed. The management authority of KUNR approved the collection of Przewalski’s horse feces samples.

### Study area

For details of the study area, please refer to Zhou et al*.*^18^. Briefly, KUNR in Xinjiang is located in the desert subregion of northwest of People’s Republic of China, which is a typical temperate continental arid climate. The composition of plants is simple and distribution is sparse. There are 49 species of national important protected wildlife living here, including Przewalski’s horse, Mongolian wild ass*,* etc.

### Collection of fecal volatiles

From May 2 to June 7, 2019, the weather conditions of sunny and windless days were selected for sample collection. We collected samples under two sets of different conditions: (1) based on ovipositing stages of *G. pecorum,* which were defined in 2019^18^, fresh feces at the stages of PREO of *G. pecorum* (May 2–8), OVIP (May 17–23), and POSO (June 1–7) were collected. (2) based on the analysis curve of moisture content for Przewalski’s horse feces over time, feces of Fresh [moisture content of feces 70–80%, i.e., OVIP mentioned above], Semi-fresh (30–40%) and Dry (≤ 10%) were collected at the oviposition stage of *G. pecorum.* The different groups of Przewalski’s horse were tracked from 11:00 am to 12:00 am (Beijing time) in the study area, and feces that were not contaminated with soil, grass and urine were randomly collected. A total of five samples (i.e., PREO, OVIP/Fresh, POSO, Semi-fresh, and Dry), each were triplicated, were collected for further analysis. Enrichment of volatiles from each fecal sample was conducted within 30 min after sampling as described below.

The collecting method of fecal volatiles was derived from Zhou et al*.*^18^ with slight improvement: feces (100.00 g) were sealed in oven bags (Reynolds, Richmond, VA). The air in the bags were removed, air filtered through activated carbon, which was used to circulate collection of volatiles without contamination, were added, so that volatiles in the bags were enriched in activated adsorption pipe (CAMSCO, Houston, TX) filled with Tenax-TA (60/80 mesh; Alltech, Deerfield, IL) by QC-1S air sampler (Beijing Municipal Institute of Labour Protection, Beijing, China). For each sample, the adsorption was conducted for 90 min at a flow rate of 0.5 L/min, and then the pipe was sealed and kept at − 20 °C for analysis by gas chromatography-mass spectrometry (GC–MS).

### Automatic thermal desorption gas chromatography–mass spectrometry analysis

The automatic thermal desorption gas chromatography–mass spectrometry analysis was derived from Zhou et al*.*^18^. In brief, the adsorption pipe was heated (260 °C for 10 min) to desorb volatiles, then they were enriched with cold-trapping (− 30 °C for 3 min) and were heated again (300 °C for 5 min) by a Turbo Matrix 650 Automatic Thermal Desorber (PerkinElmer, Waltham, MA). Subsequently, volatiles were placed into Clarus 600 Gas Chromatography (PerkinElmer) (kept 40 °C for 2 min, raised to 180 °C at 6 °C /min, then raised to 270 °C at 15 °C /min, and kept 270 °C for 3 min) and Clarus 600T Mass Spectrometry (PerkinElmer) (250 °C and 230 °C for interface and ion source temperatures in respective). The type of chromatographic column was DB-5MS UI (30 m × 0.25 mm × 0.25 μm; Agilent Technologies, Santa Clara, CA). The ionization mode of mass spectrum was electron impact ion (EI), electron energy was 70 eV, and scan range was 30–500 m/z.

### Data analysis

TurboMass 5.4.2 GC/MS software (PerkinElmer, Shelton, CT) was used to analyze volatiles. The volatile components were identified by matching their retention times, characteristic ions, and mass spectra with the NIST 08 library (National Institute of Standards and Technology, Gaithersburg, MD). Relative contents of volatiles were calculated by the area normalization method. Data were analyzed by SPSS 22.0 (IBM Corporation, Armonk, NY) and R 4.0.5 (R Core Team, R Foundation for Statistical Computing, Vienna, Austria), and diagrams were drawn by SigmaPlot 12.5 (Systat Software, San Jose, CA) and R. One-way ANOVA (least significant difference test for multiple comparisons), Independent *t*-test and Kruskal–Wallis test in the SPSS were used to conduct pairwise comparisons from relative contents of volatile components or volatile classes. The normality of distribution and homogeneity of data were examined by the Shapiro–Wilk test and Levene’s test, respectively. One-way ANOVA or Independent *t*-test was used for quantitative data. Kruskal–Wallis test was used for data that did not meet requirements for normality and homogeneity after transformation. To assess whether volatile composition was associated with different samples, NMDS analysis in R was performed. ANOSIM analysis based on Bray–Curtis similarities in R was used to test differences between volatile profiles of different samples. All statistical tests were performed at a 5% significance level, and *P*-values were adjusted for multiple comparisons of the single substances or substance classes by Benjamini–Hochberg method (controlling the false discovery rate). The data are expressed as mean ± standard error (SE)^18^.

## Supplementary Information

Supplementary Information.
