# Supplementary material for: Analysis of volatiles from feces of released Przewalski’s horse (Equus przewalskii) in Gasterophilus pecorum (Diptera: Gasterophilidae) spawning habitat
Source: Sci Rep. 2021 Aug 2;11:15671. doi: 10.1038/s41598-021-95162-9 (PMC8329074; doi:10.1038/s41598-021-95162-9)
Supplement: Supplementary file 1 — Supplementary Information. [file 41598_2021_95162_MOESM1_ESM.docx]

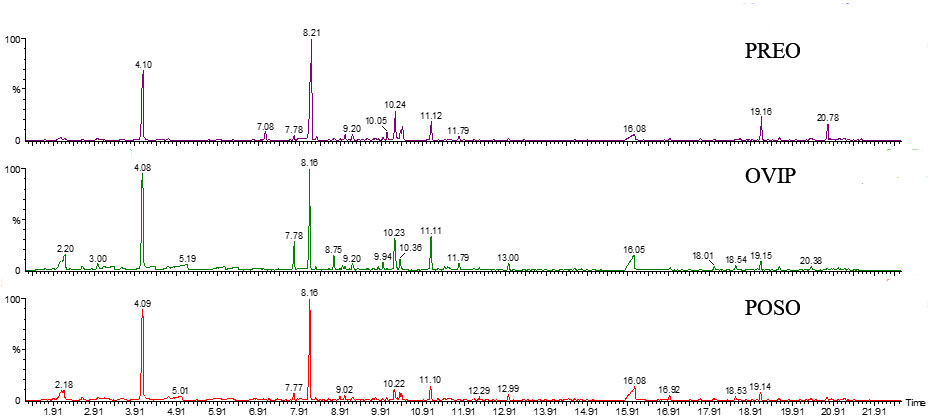


**Figure S1.** The GC/MS chromatograph of volatiles from fresh feces of Przewalski’s horse at the stages of PREO, OVIP, and POSO of *Gasterophilus pecorum.* PREO, OVIP, and POSO represent fresh feces at the stages of pre-oviposition, oviposition, and post-oviposition of *Gasterophilus pecorum*, respectively.


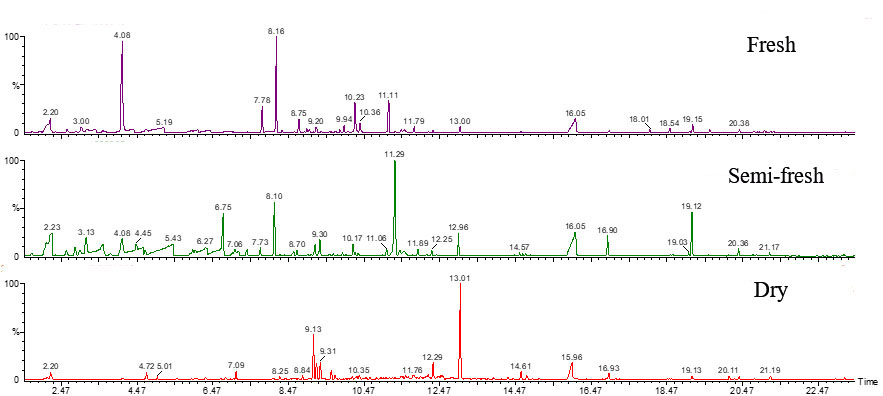


**Figure S2.** The GC/MS chromatograph of volatiles from feces of Przewalski’s horse with different freshness at the OVIP stage of *Gasterophilus pecorum.* Fresh, Semi-fresh, and Dry represent fresh, semi-fresh, and dry feces at the oviposition (OVIP) stage of *Gasterophilus pecorum*, respectively.
